# Ebi/AP-1 Suppresses Pro-Apoptotic Genes Expression and Permits Long-Term Survival of *Drosophila* Sensory Neurons

**DOI:** 10.1371/journal.pone.0037028

**Published:** 2012-05-30

**Authors:** Young-Mi Lim, Shigeo Hayashi, Leo Tsuda

**Affiliations:** 1 Animal Models of Aging, National Center for Geriatrics and Gerontology, Gengo, Obu, Aichi, Japan; 2 Laboratory for Morphogenetic Signaling, RIKEN Center for Developmental Biology, Minatojima-Minamimachi, Chuo-ku, Kobe, Hyogo, Japan; 3 Department of Biology, Kobe University Graduate School of Science, Kobe, Hyogo, Japan; University of Massachusetts Medical School, United States of America

## Abstract

Sensory organs are constantly exposed to physical and chemical stresses that collectively threaten the survival of sensory neurons. Failure to protect stressed neurons leads to age-related loss of neurons and sensory dysfunction in organs in which the supply of new sensory neurons is limited, such as the human auditory system. Transducin β-like protein 1 (TBL1) is a candidate gene for ocular albinism with late-onset sensorineural deafness, a form of X-linked age-related hearing loss. TBL1 encodes an evolutionarily conserved F-box–like and WD40 repeats–containing subunit of the nuclear receptor co-repressor/silencing mediator for retinoid and thyroid hormone receptor and other transcriptional co-repressor complexes. Here we report that a *Drosophila* homologue of TBL1, Ebi, is required for maintenance of photoreceptor neurons. Loss of *ebi* function caused late-onset neuronal apoptosis in the retina and increased sensitivity to oxidative stress. Ebi formed a complex with activator protein 1 (AP-1) and was required for repression of *Drosophila* pro-apoptotic and anti-apoptotic genes expression. These results suggest that Ebi/AP-1 suppresses basal transcription levels of apoptotic genes and thereby protects sensory neurons from degeneration.

## Introduction

Exposure to physical and chemical stresses as well as to overstimulation is a major cause of the cell death of sensory neurons and is considered a causative factor for age-related sensory dysfunction, such as age-related hearing loss (ARHL) and age-related macular degeneration (AMD) [1], [2].

A large body of evidence suggests that sensory organ defects are elicited by the overproduction of reactive oxygen species (ROS) that form in the sensory organ as a result of these insults, as well as by overstimulation by external stimuli [1], [3]. Formation of ROS triggers the transcriptional activation of genes via several distinct transcription factors, such as antioxidant-response element binding protein activator protein-1 (AP-1) [4]. AP-1 is a redox-sensitive transcription factor that participates in stress-induced apoptotic pathways in neuronal cells, as well as in normal development, by acting downstream of JNK signalling [4], [5]. Although increasing numbers of studies suggest that this transcription factor plays an important role in the survival of sensory cells, such as hair cells in the cochlea and photoreceptor cells in the retina, very little is known about how this transcription factor regulates long-term survival of sensory cells [1], [6], [7].

AP-1 acts as both an activator and repressor of transcription by forming a distinct complex with coactivators and corepressors, and this dual functioning of AP-1 occurs during many cellular events [8]–[10]. Extensive biochemical analyses have revealed that the protein complex acts as a transcriptional checkpoint for AP-1–dependent gene networks that regulate diverse biological processes, such as apoptotic processes during conditions of cellular stress [8], [9], [11]. Transducin β-like protein 1 (TBL1) and TBL1-related protein (TBLR1), two closely related F-box/WD-40–containing factors, are major components of the checkpoint machinery of AP-1, which includes the co-repressor silencing mediator for retinoid and thyroid hormone receptor (SMRT)–nuclear receptor co-repressor (N-CoR) complex [9], [12]. The *Drosophila* homologue of TBL1, *ebi*, acts as a transcriptional co-repressor by forming a complex with SMRT-related ecdysone receptor-interacting factor (SMRTER), a *Drosophila* counterpart of SMRT and N-CoR, suggesting that the co-repressor function of TBL1 may be evolutionarily conserved [13]–[16].

TBL1 was identified as a causative factor for a human age-related hearing disorder called ocular albinism with late-onset sensory neural deafness (OASD) [13], [17]. Patients with OASD have a small deletion in the TBL1 locus leading to C-terminal truncation [17]. This mutant form of TBL1 may be involved in OASD disease initiation; however, the molecular role of TBL1 in this disease remains unclear.

In this study, we found that *ebi* is required for the long-term survival of photoreceptor cells in *Drosophila*. A loss-of-function mutation in *ebi* resulted in age-related retinal degeneration. Using genetic and biochemical analyses, we found that AP-1 is involved in *ebi*-dependent photoreceptor survival. Ebi formed a complex with c-Jun, a component of AP-1, and repressed the transcription of *hid*, a major component of apoptotic gene networks [18]. Because Ebi and TBL1 are evolutionarily conserved molecules, our study of the role of *ebi* in photoreceptor survival will extend to the study of age-related sensory defects, such as age-related hearing disorders in humans.

## Results

### ebi is required for photoreceptor cell survival

**Figure 1:**
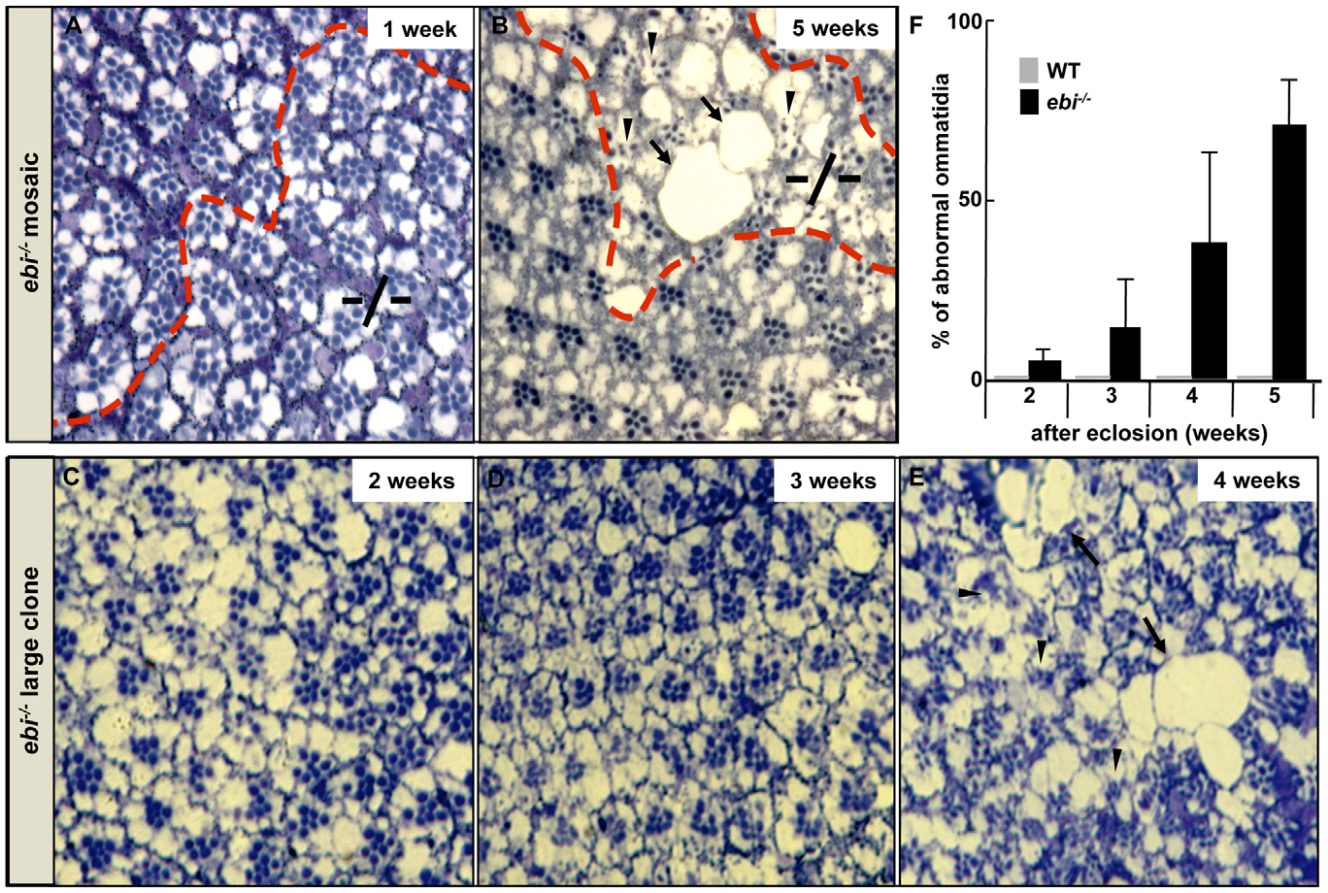
*ebi* is required for cellular survival of ommatidia. (A, B) *ey-FLP; ebi^P^, FRT40A*/+, *FRT40A* (*ebi*−/− mosaic) ommatidia 1 week (A) and 5 weeks (B) after eclosion. Note ommatidia in *ebi* mutant clones (−/−). (C–E) Ommatidia from *ey-FLP; ebi^P^, FRT40A*/*CycE^AR95^, FRT40A* (*ebi* ^−/−^ large clone) flies, in which >95% of compound eyes consist of *ebi^P^* homozygous cells, at 2 (C), 3 (D), and 4 (E) weeks after eclosion (Lee *et al.* 2001). (F) Quantification of abnormal ommatidia with reduced numbers of photoreceptor cells in *ebi* ^−/−^ clones over a 5-week period after eclosion. For comparison, wild-type (WT) ommatidia were analysed. Arrows; scar-like defects. Arrowheads; abnormal ommatidia.

Ebi is a *Drosophila* homologue of TBL1 and is involved in many biological processes [13], [15], [16], [19]. During functional analysis of *ebi*, we observed that flies that had an *ebi* mutation in their eyes showed an age-dependent loss of eye pigment (Figure S1). The loss of eye pigment is sometimes linked to cellular degeneration in compound eyes [20]. We next observed the phenotype of somatic clones of the *ebi* mutant (*ebi* ^−/−^) [21]. One week after eclosion, homozygous *ebi* ^−/−^ (−/−) cells formed ommatidia with a near-normal set of photoreceptors (Figure 1A). At 5 weeks, however, *ebi* ^−/−^ (−/−) cells showed a severely degenerated phenotype in a cell-autonomous manner (Figure 1B). Several scar-like defects appeared in the retinas (Figure 1B, arrows) along with abnormal ommatidia with a reduced number of photoreceptor cells (Figure 1B, arrowheads). Quantification of the number of abnormal ommatidia with a loss of rhabdomeres in large *ebi* ^−/−^ mosaic clones (referred to as *ebi* ^−/−^ clones) revealed that severe degeneration occurred at 4 weeks after eclosion (Figure 1C–F) [22]. These data suggest that *ebi* itself is required for the long-term survival of sensory cells.

### C-terminal truncation of Ebi causes age-related retinal degeneration

**Figure 2:**
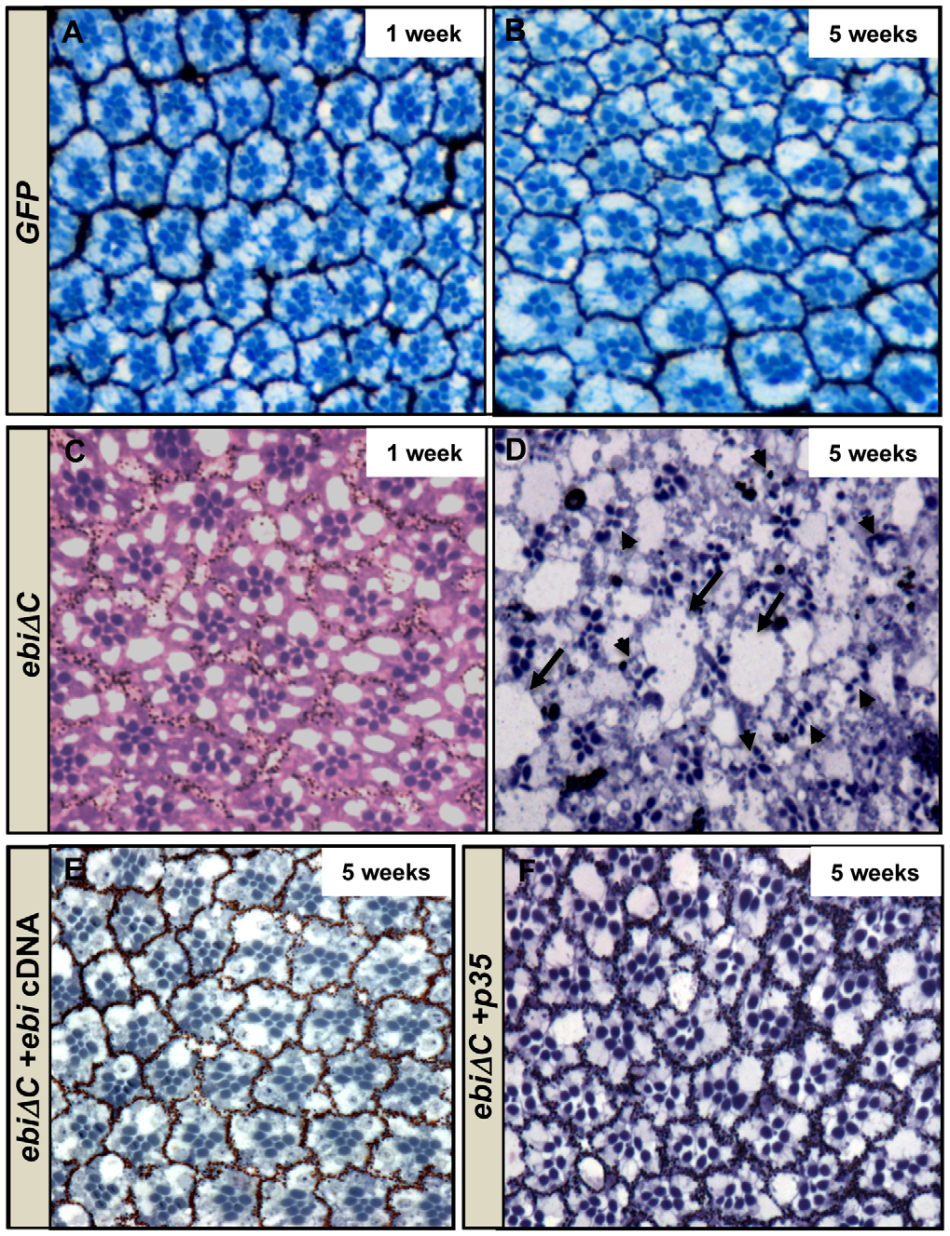
C-terminal truncation of Ebi causes age-related retinal degeneration. (A, B) *GMR-GFP/+* (*GFP*) ommatidia 1 week (A) and 5 weeks (B) after eclosion. (C, D) *GMR-ebiΔC*/+ (*ebiΔC*) ommatidia 1 day (C) and 5 weeks (D) after eclosion. Arrows; scar-like defects. Arrowheads; abnormal ommatidia. (E) *GMR-Gal4*/+*; GMR-ebiΔC*/+; *UAS-ebi*/+ (*ebiΔC*+*ebi*) ommatidia, which express *ebi* cDNA via *GMR-Gal4*, 5 weeks after eclosion. (F) *GMR-Gal4*/+; *GMR-ebiΔC*/+; *UAS-p35*/+(*ebiΔC*+*p35*) ommatidia. *p35* inhibited *ebiΔC*-induced retinal degeneration.

TBL1 may be required for the long-term survival of sensory neurons in the cochlea, because patients with OASD have a mutation in the TBL1 locus that leads to a small deletion and produces a mutant form of TBL1 in which the C-terminal half, including some of the WD40 repeats, is deleted (Figure S2) [17]. To elucidate the consequences of this C-terminal mutation in TBL1, we introduced a similar truncation mutation in *ebi* and expressed it in the eye (*GMR-ebiΔC*) (Figure S2) [13]. Control *GMR-GFP/+* flies (referred to as *GFP*) did not show degenerate phenotype five weeks after eclosion (Figure 2B compare with 2A). After eclosion, *GMR-ebiΔC*/+ flies (referred to as *ebiΔC*) also possessed a near-normal complement of photoreceptors in each ommatidium (Figure 2C, compare with 2A). Five weeks later, however, those retinas were severely degenerated (Figure 2D). This retinal degeneration phenotype was completely rescued by increased dosage of wild-type Ebi (Figure 2E), suggesting that the C-terminal truncation of Ebi is a dominant-negative mutation for *ebi* itself.

To observe the degenerative phenotype in more detail, we introduced a baculovirus p35, a caspase-inhibitory protein, and found that the degeneration phenotype of *ebiΔC*-expressing retinae was suppressed by this treatment (Figure 2F) [23]. This result suggests that *ebi* has an anti-apoptotic function.

### Jra shows genetic and physical interactions with Ebi

**Figure 3:**
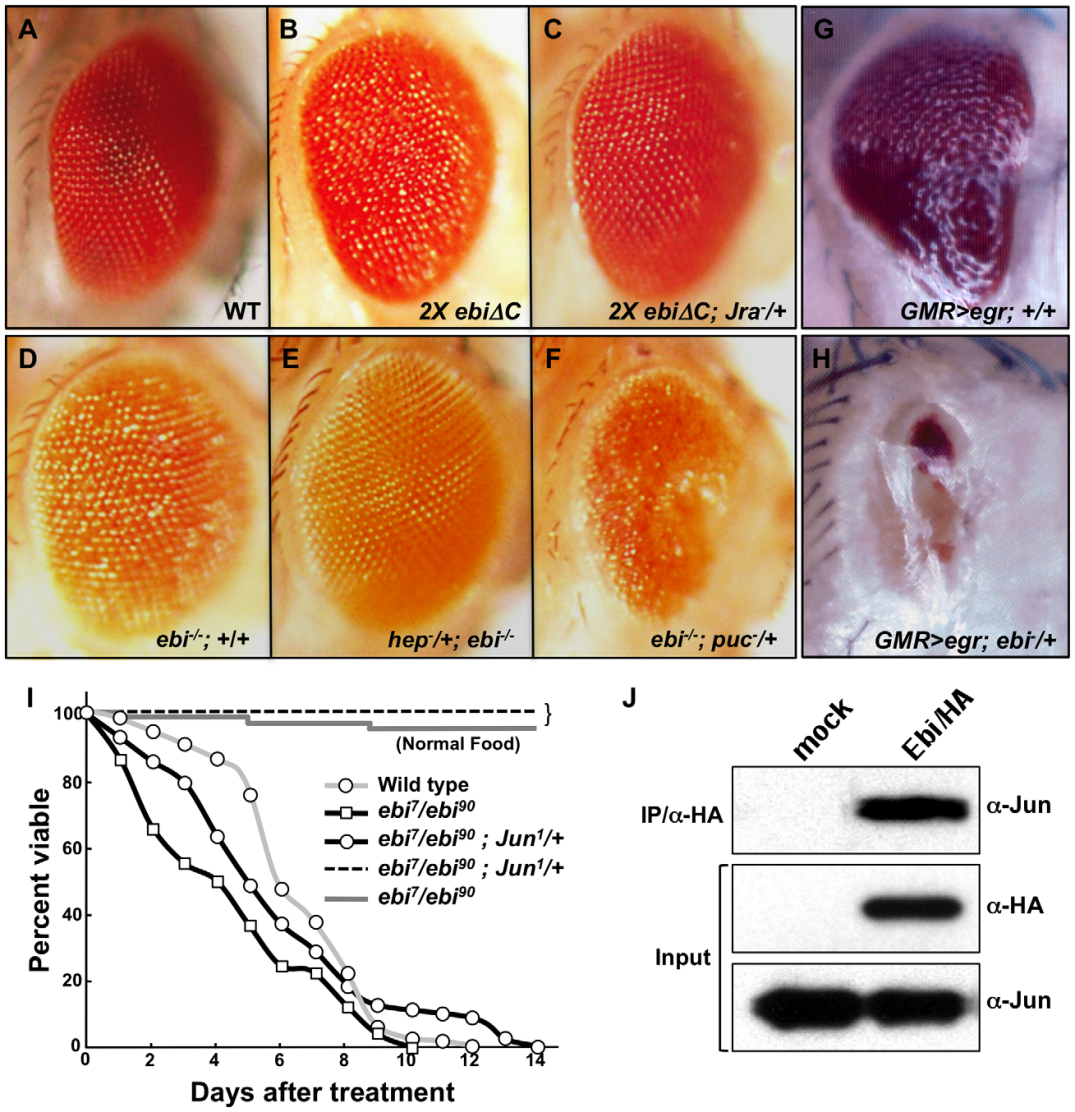
*ebi* interacts with *Jra* and antagonizes *JNK* signaling. Compound eyes from wild type (A), *GMR-ebiΔC, GMR-ebiΔC*/+ (*2×ebiΔC*) (B), *GMR-ebiΔC, GMR-ebiΔC*/*Jra^1^* (*2×ebiΔC; Jra^1^*/+) (C), *ey-FLP; ebi^P^, FRT40A*/*CycE^AR95^, FRT40A* (*ebi* **^−/−^**) (the same genotype as in Figure 1C–E) (D), *hep^r75^*/*ey-FLP; ebi^P^, FRT40A*/*CycE^AR95^, FRT40A* (*hep^r75^*/+; *ebi* **^−/−^**) (E), and *ey-FLP; ebi^P^, FRT40A*/*CycE^AR95^, FRT40A; puc^E68^*/+(*ebi* **^−/−^**; *puc^E68^*/+) (F). (G, H) *ebi* showed a strong genetic interaction with *TNF-α.* (G) *GMR-Gal4*/+; *egr^GS11687^*/+, *TNF-α* overexpression induced a small-eye phenotype (Igaki *et al.* 2002). (H) *GMR-Gal4*/+; *egr^GS11687^*/*ebi^4^*, in which a copy of *ebi* was removed, enhanced the eye phenotype. (I) Lifespan analysis for wild type and for strains containing *ebi* mutants (*ebi^7^*/*ebi^90^*) and *ebi* mutants with a copy of *Jun* removed (*ebi^7^, Jra^1^*/*ebi^90^*, +) cultured with or without 7.5 mM paraquat. Wild-type flies died within 12 days (open circle and gray line). Strong enhancement occurred in *ebi* mutant escapers (open square and black line). Removing a copy of *Jun* suppressed the sensitivity to paraquat (open circle and black line). The median life span was 5.9 d, 4.2 d, and 5.1 d for wild-type, *ebi* mutant, and *ebi* mutant with *Jun* males, respectively. Statistical analysis was performed using the log rank test. For wild type versus *ebi* mutant, p<0.001. For *ebi* mutant versus *ebi* mutant with a copy of *Jun* removed, p<0.001. n = 100. (J) Immunoprecipitation assay. S2 cells were transfected with a *HA-ebi* expression vector, and proteins were immunoprecipitated with hemagglutinin antibody and immunoblotted as indicated.

To elucidate the molecular mechanism of *ebi*-mediated photoreceptor survival, we performed a screen for a genetic suppressor of *GMR-ebiΔC*. Although one copy of *GMR-ebiΔC* produced a slightly abnormal rough-eye phenotype, two copies of *GMR-ebiΔC* (referred to as *2×ebiΔC*) produced a severe rough-eye phenotype (Figure 3A and 3B) [13]. To identify genetic modifiers for *2×ebiΔC*, we performed genetic interactions using a deficiency chromosome and found that *Df(2R)X1*, which has a deletion between 46C and 47A1 in the chromosomal region, suppressed the rough-eye phenotype when it was trans-heterozygous with *2×ebiΔC* (data not shown). We then searched for the mutation that is a causative factor for this suppression within this chromosomal region (46C–47A1). From this analysis we found that a single copy of *Jun-related antigen* (*Jra*), a *Drosophila* homologue of *c-jun* that acts downstream of *JNK* signalling, strongly suppressed the rough-eye phenotype when it was trans-heterozygous with *2×ebiΔC* (Figure 3C).

We found that *ebi* shows a genetic interaction with the JNK signalling pathway as well as with *Jra* under different conditions. Large clones of *ebi* (*ebi* ^−/−^) showed a mild rough-eye phenotype (Figure S1; Figure 1C–E; Figure 3D). We introduced a *hemipterous* (*hep*; a kinase activator of JNK) mutation into this background and found that *hep* suppressed the rough-eye phenotype of *ebi* ^−/−^ (Figure 3E). In contrast, *puckered* (*puc*; a phosphatase acting on JNK) enhanced the rough-eye phenotype (Figure 3F) [24]. Furthermore, removing one copy of *ebi* strongly enhanced the small-eye phenotype that was mediated by overactivation of JNK signalling via overexpression of *eiger* (*egr*), a TNF superfamily ligand that activates JNK signalling cascades (Figure 3G and 3H) [25]. In this case, however, we also observed strong enhancement of *egr* induced small eye phenotype by overexpressing *ebi* (Figure S3). We presume this might be due to the inhibition of the proper Ebi function by overexpressing *ebi* in this sensitized background. We also observed that survival of *ebi* mutant escaper flies was very sensitive to stimulation by ROS, and this seemed to be *Jra* dependent (Figure 3I). These results suggest that *ebi* acts antagonistically to JNK signalling pathway.

Jra and Fos form part of the activator protein 1 (AP-1) complex, which acts downstream of JNK signalling, and Ebi and Jra are localized in the nucleus [19], [26]. Therefore, we expected to observe a physical interaction between these transcriptional regulators. We confirmed that endogenous Jra was co-immunoprecipitated by epitope-tagged Ebi in cultured S2 cells (Figure 3J).

The ability of Ebi to form a complex with AP-1 and SMRTER, the *Drosophila* counterpart of mammalian N-CoR/SMRT, parallels that of mammalian TBL1, suggesting that this interaction is evolutionarily conserved [14]–[16].

### ebi negatively regulates hid expression

**Figure 4:**
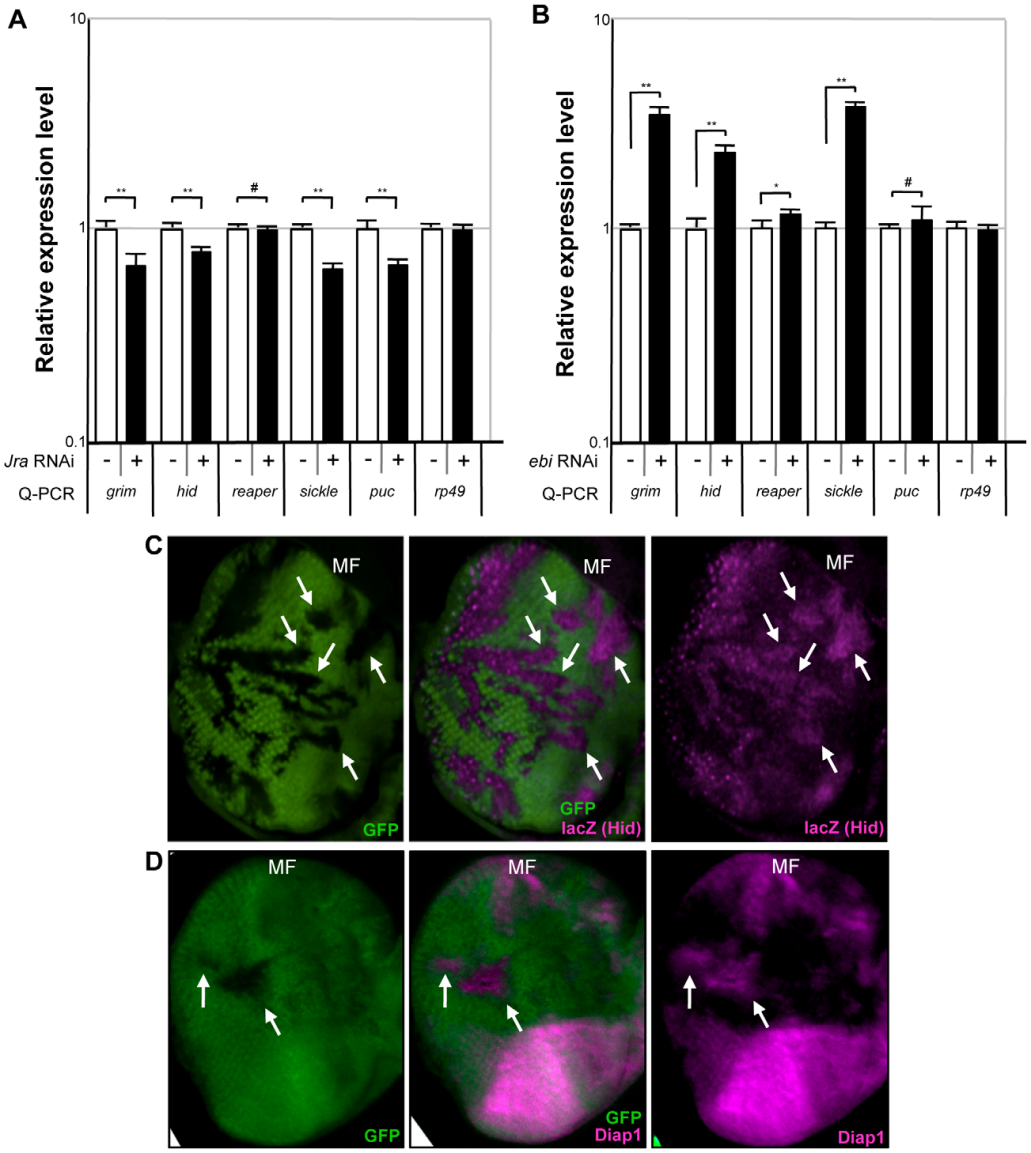
*ebi* antagonizes *hid* expression. (A, B) Quantitative real-time PCR (qRT-PCR) analysis were performed using specific primers. Each mRNA from S2 cells treated with dsRNA against *Jra* (A) or *ebi* (B) was used for the analysis. Data are shown as the mean ± SEM. * = p<0.05; ** = p<0.01; # = p>0.05. (C) Mosaic clones of *ebi^P^*. Eye discs from *ey-FLP; ebi^P^, FRT40A*/*ubi-GFP, FRT40A; hid^05014^*/+ were stained with anti-lacZ and visualized for GFP expression. *ebi* mutant clones showed slightly increased *hid* expression. MF: morphogenetic furrow. (D) Mosaic clones of *ebi^E4^*. Eye discs from heat-induced *hs-FLP; ebi^E4^, FRT40A*/*ubi-GFP, FRT40A* were stained with anti-Diap1 and visualized for GFP expression. Diap1 was increased in *ebi* mutant clones.

In addition to acting as a transcriptional activator, AP-1 can repress transcription in some cellular contexts [8]–[10], [27]. Our studies suggested that Ebi may repress apoptosis pathways by forming a complex with AP-1. Notably, previous reports showed that JNK and AP-1 positively regulate expression of the pro-apoptotic gene *hid* in *Drosophila* [28], [29]. Therefore, we reasoned that AP-1 has a dual function and that the Ebi/AP-1 complex may act as a transcriptional repressor of the expression of *hid*. To determine whether *hid* is a target gene of *Jra*, we performed quantitative PCR (qPCR) analysis in S2 cells. We first confirmed that reduction of the activity of *Jra* with RNAi decreased the expression of *puc*, a typical JNK/AP-1 target gene (Figure 4A; Figure S4). Reducing the activity of *Jra* with RNAi also decreased the expression of *hid* (Figure 4A; Figure S4). The expression level of *hid* was, however, increased by treating the cells with RNAi against *ebi* (Figure 4B; Figure S4). We found that *grim*, *sickle* and *reaper*, which are also pro-apoptotic genes, were also increased when *ebi* expression was reduced, suggesting that *ebi* downregulates most of pro-apoptotic genes expression (Figure 4B). We confirmed that the expression level of *puc* was not increased by treatment with *ebi* dsRNA, suggesting that JNK itself may not be activated in these situations (Figure 4B; see Discussion).

To confirm whether *ebi* downregulates *hid* expression *in vivo*, we performed mosaic analysis. Mosaic clones of a strong loss-of-function allele of *ebi* in the developing eye discs indeed showed increased expression of *hid-lacZ*, supporting that *ebi* may play an inhibitory role against *hid* expression (Figure 4C, arrows). However, we could not detect severe apoptotic phenotype during larval development of *ebi* mutants (Figures 1 and 2). We have analyzed the expression level of *DIAP1*, a *Drosophila* homologue of *inhibitor of apoptotis*, and found that the expression level of DIAP1 was increased in *ebi* mutant clones (Figure 4D). We presume that increasing expression level of DIAP1 in *ebi* mutant might contribute for the protection of retina from degeneration at the early stage of development.

### ebi represses hid expression via the AP-1 target site in the hid promoter

**Figure 5:**
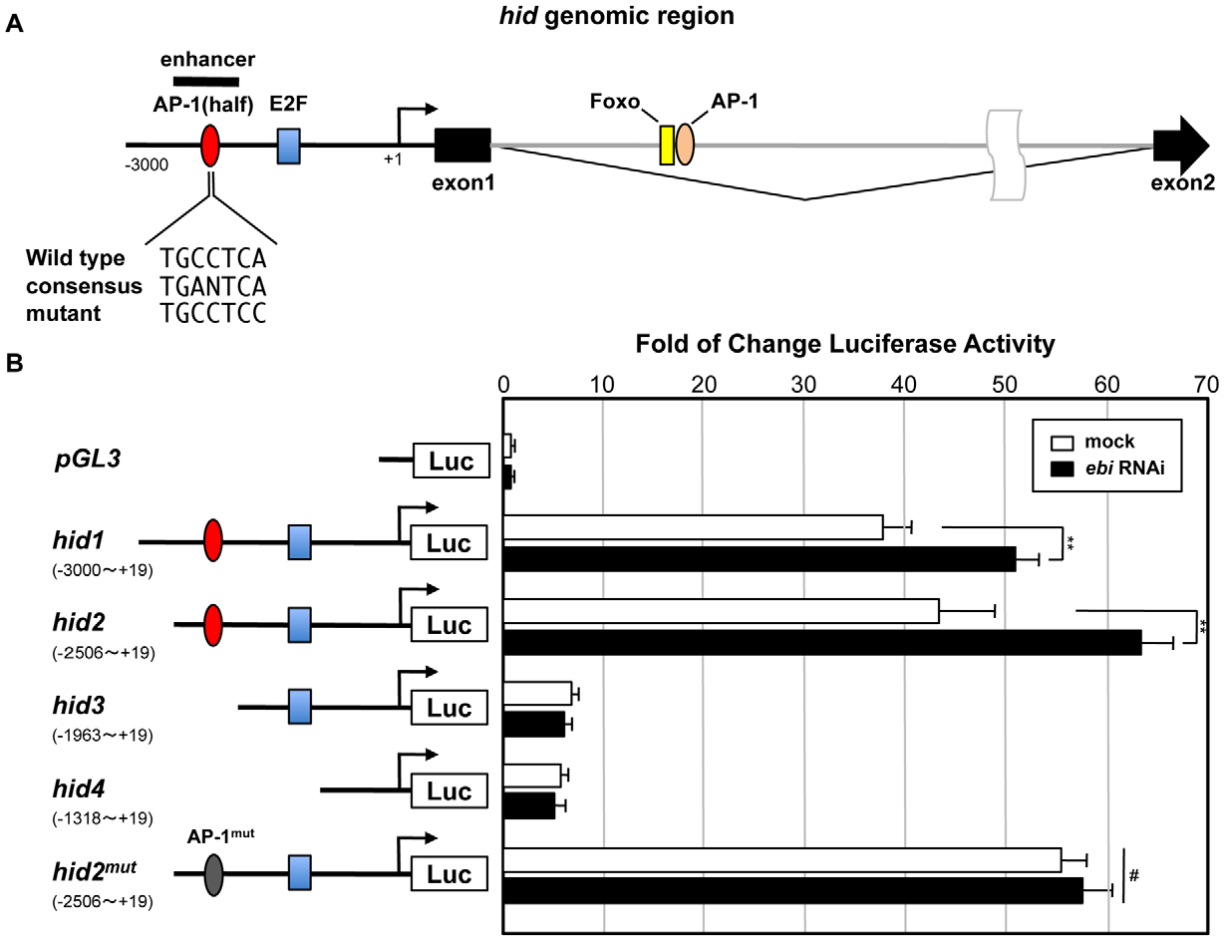
*ebi* downregulates *hid* expression via the AP-1 target site in the promoter region of *hid*. (A) Schematic of the promoter region of *hid*. The AP-1 half site and its mutant forms are indicated. (B) Luciferase constructs with different lengths of the promoter region of *hid*. Effects of *ebi* dsRNA on expression of the wild-type or mutant forms of *hid*-*luc* are shown in the graph. ** = p<0.01; # = p>0.05. Data are shown as the mean ± S.E.M.

Extensive analysis of the transcriptional regulation of *hid* has been reported. Foxo and AP-1 regulate *hid* expression via specific binding sites in the first intron of *hid*, whereas the E2F-binding site in the upstream region of the *hid* transcriptional start site is used in response to stress-induced signalling (Figure 5A) [29], [30]. To elucidate the site(s) required for transcriptional repression of hid by Ebi, we performed reporter assay using the upstream regulatory region of hid (Figure 5A). We made several reporter constructs that contain different lengths of the promoter region of hid followed by the luciferase gene and determined whether the level of ebi expression affected promoter activity (Figure 5A). We found an apparent enhancer for hid expression at the 5′ distal region of the E2F-binding site and showed that this region was sensitive to ebi RNAi (Figure 5A and 5B). We identified one atypical binding consensus sequence for AP-1 (AP-1 half site) in this region (Figure 5A). A point mutation in the AP-1 half site of the reporter resulted in resistance to ebi RNAi (Figure 5A and 5B). In this case, however, the basal level of the reporter activity was also increased, suggesting that the Ebi/AP-1 complex represses the expression of hid through the AP-1 site (Figure 5A and 5B; see Discussion).

### Jra is required for ebi-dependent long-term survival of photoreceptor cells

**Figure 6:**
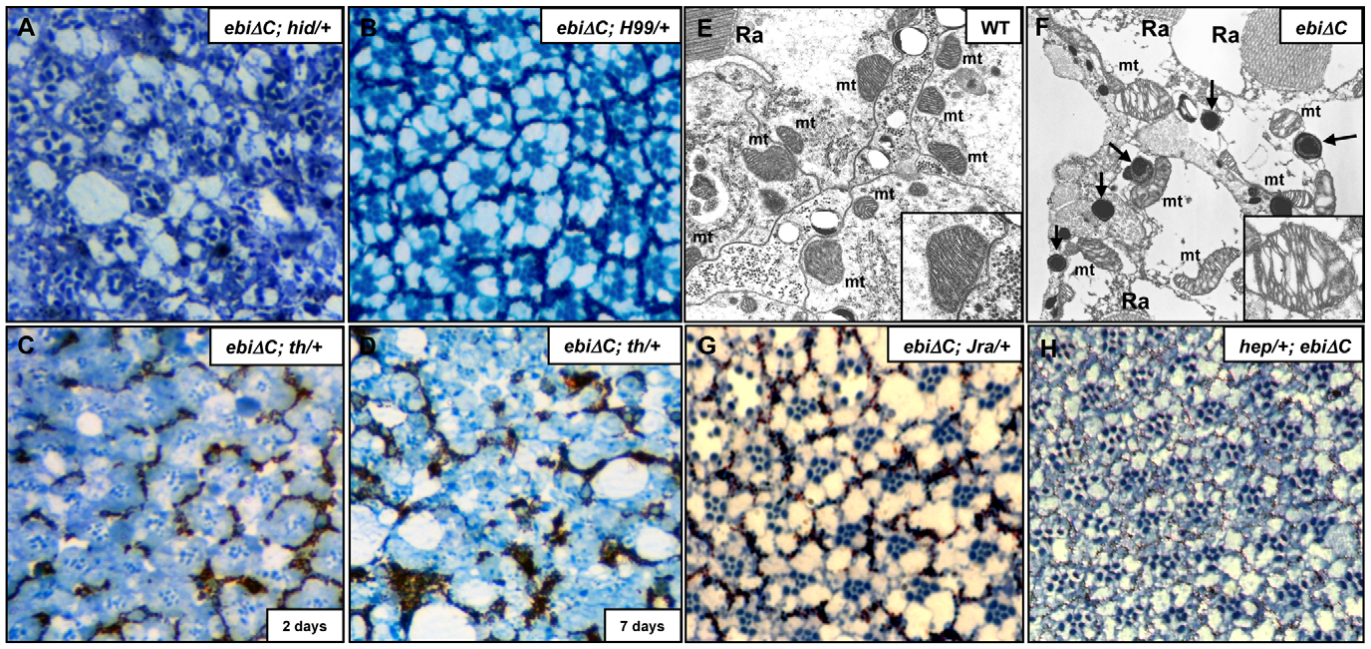
*Jra* is required for *ebi*-dependent retinal degeneration. Eye sections of aged flies. (A) *GMR-ebiΔC*/*+; hid^05014^/+, (ebiΔC; hid*/+*)* ommatidia. 5 weeks after eclosion. (B) *GMR-ebiΔC/+; Df(3L)H99/+, (ebiΔC; H99/+)* ommatidia. 5 weeks after eclosion. (C, D) *GMR-ebiΔC*/*+; th^4^/+, (ebiΔC; th*/+*)* ommatidia. Eye sections of 2 days aged flies showed slight degeneration phenotype (C). One week aged flies showed severe degeneration phenotype (D). (E, F) Transmission electron microscope images of ommatidia. 5 weeks after eclosion. Wild-type flies had normal ommatidia, and each mitochondrion was arranged along the cell-cell junction with compact structures (E, inset). *ebiΔC* flies, in contrast, had electron-dense vesicles (arrows) and showed mitochondrial swelling and changes in mitochondrial cristae (F, inset). mt, mitochondrion; Ra, rhabdomere. (G) *GMR-ebiΔC*/*Jra^1^, (ebiΔC; Jra*/+*)* ommatidia. 5 weeks after eclosion. (H) *hep^r75^*/+; *GMR-ebiΔC*/+, *(hep*/+; *ebiΔC)* ommatidia. 5 weeks after eclosion.

In ebi mutant retinal cells, upregulation of hid was observed in late third instar larvae (Figure 4C), but cell death occurred mainly in aged adults (Figure 1C–F). We crossed *ebiΔC* with *hid* and found that *hid* mutation could not rescue the late-onset retinal degenerative phenotype of *ebi* mutant (Figure 6A). These suggest that an additional cell death stimulus must be involved in this situation. Then, we crossed *ebiΔC* with a small deletion mutant of pro-apoptotic genes locus, which deletes all of the pro-apoptotic genes, and found that the degeneration phenotype was suppressed by this treatment (Figure 6B). This indicates that increasing expression level of pro-apoptotic genes might contribute for the late-onset degeneration. To confirm this idea we crossed *ebiΔC* with *thread* (*th*: mutant form of *Diap1*). Although *th* showed very mild effect on the compound eye phenotype along with *ebiΔC*, it severely enhanced retinal degeneration of *ebiΔC* at the adult onset (Figure 6C and 6D; Figure S5). These results suggest that multiple pro-apoptotic genes might be required for the late-onset degeneration phenotype in *ebi* mutant.

In the previous study it has been shown that increasing expression level of both *hid* and *reaper* resulted in changes in mitochondrial ultrastructure and caused apoptosis [31]. To see the structure of mitochondria in aged *ebiΔC* (5 weeks after eclosion), we used transmission electron microscopy. Compared with the wild type, *ebiΔC* showed defective mitochondria with swollen and abnormal cristae in the photoreceptor cells (Figure 6E and 6F).

Given the fact that most of pro-apoptotic genes expression seemed to be regulated by *Jra* and *ebi* (Figure 4A and 4B), we expect that JNK/AP-1 pathway regulate the expression of pro-apoptotic genes expression and is required for long-term survival of ommatidia. To elucidate this possibility, we tested the role of signalling molecules in the JNK pathways. We crossed *ebiΔC* with *Jra* or *hep* and found that *Jra* and *hep* mutations consistently suppressed the late-onset retinal degenerative phenotype of *ebiΔC* (Figure 6G and 6H). These data suggest that retinal cells survival might be maintained by the activity of the Ebi/AP-1 repressor complex acting downstream of JNK signalling.

**Figure 7:**
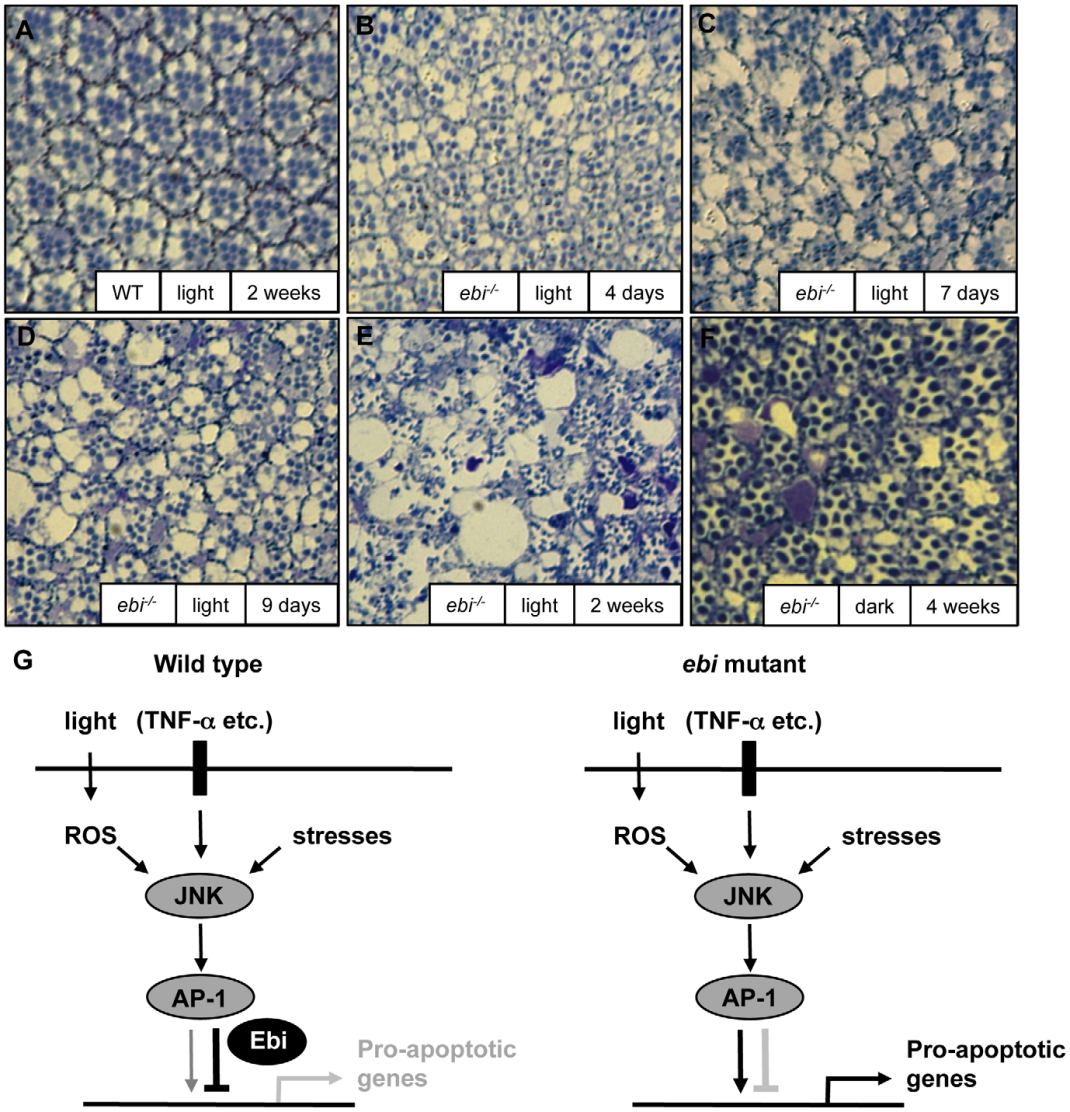
Light-dependent retinal degeneration in an *ebi* mutant. (A–D), Flies were raised under constant illumination (A–E) or darkness (F) at the times shown. Wild-type (A) and *ey-FLP; ebi^P^, FRT40A/CycE^AR95^, FRT40A* (*ebi^P^* large clone) (B–F) ommatidia are shown. (G) Models of *ebi* activity in photoreceptor cells survival.

There is compelling evidence that exposure of the eye to visible light creates photo-oxidative stress, which leads to oxidative damage of photoreceptors [32]. We thus considered light exposure as a potential trigger for age-related retinal degeneration mediated by *ebi*. Flies with *ebi* mutant retinae that were cultured under constant illumination showed severe degeneration by 14 days after eclosion (Figure 7A–E, compare with Figure 1C), whereas wild-type ommatidia did not show any structural changes under the same conditions (Figure 7A). In contrast, when mutant flies were raised under constant darkness, the eye phenotype remained mild 4 weeks after eclosion (Figure 7F, compare with Figure 1E). Similar light dependency of the retinal degeneration phenotype was observed with *ebiΔC* flies (Figure S6). These results suggest that light exposure critically affects the onset of retinal degeneration associated with *ebi* mutants.

## Discussion

TBL1 was originally reported as a homologous molecule of *ebi*, a downstream component of epidermal growth factor receptor signaling in *Drosophila* [13]. Subsequently, TBL1 and a related molecule, TBLR1, were identified as components of the N-CoR/SMRT/HDAC3 repressor complex. TBL1 and TBLR1 were shown to act in many aspects of developmental processes by forming a complex with different types of transcription factors, including nuclear receptor molecules and AP-1 [9], [12]. AP-1 is a dual-functional transcription factor that acts as either a transcriptional activator or repressor by forming a complex with co-activators or co-repressors [9], [27]. Because Ebi also seems to act as a co-repressor by forming a complex with SMRTER, and Ebi and AP-1 form a complex and are required for transcriptional repression, therefore, the regulatory system of AP-1, including its co-repressor mechanism, may be evolutionarily conserved [14], [15] (Figures 3,4, and 5).

Our data suggest that the Ebi/AP-1 co-repressor complex represses the expression of *hid* via an AP-1 target sequence that is located far upstream of the E2F-binding site (Figure 5A). A previous report, however, showed that the AP-1 binding site in the first intron is a positive regulatory site for *hid* expression (Figure 5A) [29]. We predict that multiple AP-1 sites in *hid* may contribute differently to the regulation of *hid* expression. A recent study has shown that expression of some AP-1 target genes may be regulated through multiple AP-1 recognition sites around the genes [33], thus, the contribution of each AP-1 target site to the transcriptional output seems to be dependent on the environment around the occupancy site. In support of this notion, *ebi* did not seem to act as a transcriptional repressor of *puc*, despite the fact that *puc* is a typical AP-1 target gene of JNK/AP-1 signaling (Figure 4B) [24].

TBL1 was also identified as a causative factor of OASD [17]. In patients with OASD, hearing decreases around age 40, suggesting that TBL1 may be required for sensory cell survival. In patients with OASD, about half of the C-terminal WD40 repeats in TBL1 are truncated and replaced with a nonsense peptide sequence (Figure S2). It is not clear whether the C-terminal truncation of TBL1 causes the disease or if the fusion peptide itself has an extra function. Therefore, it is important to determine if simple C-terminal truncation of Ebi affects the survival of sensory neurons in the fly system. Our data clearly suggest that *ebi* is required for the survival of photoreceptor neurons and that simple C-terminal truncation of Ebi had a dominant-negative effect on the survival (Figures 1 and 2). In support of these results, recent structural analysis of TBL1 revealed that the N-terminal region of this molecule seems to act as an assembly site for the formation of a TBL1 tetramer, suggesting that C-terminal truncation of this molecule may specifically inhibit TBL1 activity [34], [35]. These observations lead to the conclusion that C-terminal truncation of TBL1 in patients with OASD creates a dominant-negative TBL1 that inhibits the survival of sensory hair cells.

Apoptotic cell death has been implicated in age-related sensory defects, such as ARHL and AMD [36]–[38]. Several signaling pathways regulate apoptotic induction in sensory cells [38], [39]. Among the downstream executors of apoptotic signaling pathways, apoptosis-related molecules, including Bax and p53, have been shown to be upregulated in sensory cells in mouse models of ARHL and AMD [36], [40]. Therefore, the expression level of apoptosis-related genes may be an important issue for disease formation. Many molecules regulate the expression of apoptosis-related genes in cells. Notably, mice lacking *junD* show enhanced levels of Bax and p53, suggesting that AP-1 is a key regulatory molecule for maintaining low expression of apoptosis-related genes [41]. Our mosaic analysis using third instar larval eye discs showed that *hid* expression was increased in *ebi* mutant clones, suggesting that the Ebi/AP-1 repression system may be involved in regulating the basal expression level of the pro-apoptotic gene *hid* in sensory neurons (Figure 4C). In this situation, however, we noticed that adult flies with the *ebi* mutation did not show prominent apoptotic phenotypes until around 4 weeks after eclosion (Figure 1C–F). We found that anti-apoptotic molecule DIAP1 was increased in *ebi* mutant clones, suggesting that *hid* expression was not enough to induce apoptosis during development (Figure 4D). To support this idea mutation of *Diap1* enhanced the retinal degeneration phenotype in adult stage (Figure 6C and 6D). We believe that this adult-onset apoptotic phenotype in *ebi* may indicate that apoptotic induction in the *ebi* mutant is required for genes in addition to *hid* at the adult stage. In this study we have found that the retinal degeneration in *ebi* mutant was dependent on the light exposure (Figure 7A–E). However, complete darkness still induced late-onset retinal degeneration, suggesting that there might be additional cue to induce late-onset retinal degeneration (Figure 7F). We presume aging processes might be involved in this situation, since growing number of evidences are suggesting that ROS and aging processes are tightly related to maintain animal life span [42]. In the future, we hope to reveal how aging processes affect the apoptotic phenotype induced by the *ebi* mutation.

Taken together, our results demonstrate that *ebi* is required for the long-term maintenance of sensory and supporting cells in ommatidia and that *ebi* acts by antagonizing AP-1. Our data suggest that simultaneous suppression of ROS stimulation and pro-apoptotic gene expression, both of which are mediated by Ebi and AP-1, reduce the baseline level of apoptotic signalling and facilitate long-term survival of photoreceptor neurons (Figure 7). Reducing the activity of *ebi* may cause over-stimulation of apoptotic signalling and induce target gene expression (Figure 7). We suggest that the TBL1/AP-1 complex plays an analogous role in vertebrates to protect sensory neurons from stress-induced apoptosis to facilitate the long-term function of auditory neurons, which must last for the lifetime of an organism.

## Materials and Methods

### Drosophila stocks and genetics

The following stocks were used in this study: *Oregon-R* as wild type, *GMR-ebi*, *GMR-ebi^DN^* (referred to as *GMR-ebiΔC*), *ebi^4^*, *ebi^90^*, *ebi^P^*, *ebi^P7^*, and *ebi^11^* [13], [15]; *y^1^w^1118^*; *Jra^1^*; *GS11687(eiger); GS1226(eiger)*. *GMR-p35*, *GMR-Gal4* (Hay *et al*, 1994), and *cycE^AR95^FRT40A*/*CyO* were described previously (Lee *et al*, 2001). *Df(3L)H99, kni[ri-1] p[p]/TM3, Sb[1]* was obtained from Bloomington Drosophila Stock Center. *w, hep^r75^*/*FM7*, *puc^E69^*/*TM6B*, and *hid^05014^*/*TM3* were provided by Dr. T. Adachi-Yamada. *UAS-GFP* and *th^4^/TM6B* were provided by Dr. M. Miura. The deficiency kit was provided by the Kyoto Stock Center, Japan.

Clones of homozygous *ebi* mutant cells were generated by eye-specific expression of the *FLP* recombinase using the *eyeless* (*ey*) promoter or heat induced recombinase in strains bearing *ubi-GFP* marked *FRT* chromosomes [21].

### Histochemistry

Immunostaining of imaginal discs was done as described [16]. The following antibodies were used in this study: mouse anti–β-galactosidase (1∶100; Developmental Studies Hybridoma Bank), anti-Diap1 antibody (1∶100; kindly provided by Dr. M. Miura) and a Cy3-conjugated secondary antibody (1∶200; Chemicon). Samples were mounted using VectaShield (Vector) and observed using Fluoview 500 (Olympus) microscopes.

Plastic sectioning of eyes was carried out as described [13]. Transmission electron microscopy was performed by Hanaich Corp.

### S2 cell experiments

*Drosophila* Schneider cells (S2 cells) [16] were cultured in Schneider's Insect Medium (Gibco) containing 10% fetal bovine serum and antibiotics at 25°C. pUAST-vectors were cotransfected with actin5C-Gal4 drivers using Effctene transfection reagent (Qiagen), according to the manufacturer's instruction, and the cells were cultured for 2 days after transfection before next treatment. For the RNAi experiments, we followed the protocol described before [43].

### Luciferase assay

A reporter construct for *hid* or a mutant form of *hid* was established by introducing the promoter construct (from genomic DNA) into the *pGL3-Basic* vector (Promega). Promoter constructs were transfected into S2 cells (1×10^6^ cells) using Effectene Reagent (Qiagen), together with *pActin-RL* in the presence or absence of dsRNA. After 48 h, the cells were lysed, and firefly luciferase activity was analyzed with the dual luciferase reporter assay system (Promega). *Renilla* luciferase activity was used to normalize luciferase activity in each sample.

### Oxidative stress treatment

Flies were starved in empty vials for 3 h and then transferred to vials containing a gel of phosphate-buffered saline, 10% sucrose, 0.8% low-melt agarose, and 7.5 mM paraquat, which was added to the solution after cooling to 40°C. A control population of flies was placed in vials containing the phosphate-buffered saline–sucrose gel without paraquat. Dead flies were counted every 24 h (*n* = 90 for each gender and genotype). Each paraquat experiment was done in triplicate.

### Immunoprecipitation

Immunoprecipitation and S2 cell transfection experiments were performed essentially as described [15]. The following antibodies were used in this study: rabbit anti-Jun (Santa Cruz Biotech.) and rat anti-HA (3F10, Roche).

### Real-time quantitative PCR analysis

Total RNAs were isolated from the first instar larvae, third instar larvae, adult flies, or S2 cells using RNA purification kit (Quiagen), and each cDNAs were synthsized with Primescript RT reagent (TAKARA) using oligo-dT as a primer. mRNA was quantified using a Thermal Cycler Dice Real Time System (TAKARA) with SYBR Premix Ex Taq (TAKARA). Obtained data were normalized using *rp49* mRNA level as a control. The thermal cycling parameters were as follows: 40 cycles of 95°C for 10 s, 60°C for 30 s. At least three independent experiments were performed each data analysis.

### Continuous light and dark treatment

Flies were placed under a light source (fluorescent lamp). The distance from the light source to the flies was 15 cm to the bottom of the cotton plug and 18 cm to the top of the food. For constant darkness, flies were placed in a dark room. The vials were changed every 3–4 days at 25°C so that the environment remained as constant as possible.

Additional Materials and Methods are described in Methods S1.

## Supporting Information

Figure S1 **Age-dependent pigment loss in** ***ebi*** **mutant eyes.** (A) Wild-type adult fly 5 weeks after eclosion. (B–D) *ey-FLP; ebi^P^, FRT40A*/*CycE^AR95^, FRT40A* (*ebi^P^* clone). After 2 weeks, eye pigment was retained (B). After 4 weeks, however, eye pigment was reduced (arrows; C). Severe pigment loss was observed after 6 weeks (D).
(TIF)

Figure S2 **Structures of wild-type and mutant versions of TBL1 and Ebi.** TBL1 from patients with OASD is truncated (TBL1^OASD^) because of a small deletion in the genomic DNA [17]. EbiΔC is a truncated form of Ebi that results in the deletion of the C-terminal WD40 repeats [13].
(TIF)

Figure S3 **Overexpression of** ***ebi*** **enhanced** ***egr*** **induced small eye phenotype.** *GMR-Gal4*/+; *egr^GS11687^*/+, *TNF-α* overexpression induced a small-eye phenotype. *GMR-Gal4*/+; *egr^GS11687^*/GMR-*ebi*, in which *ebi* was overexpressed under glass promoter, enhanced the eye phenotype.
(TIF)

Figure S4 **The efficiency of knock-down in** ***Jra*** **and** ***ebi*** **RNAi experiment.** Western blot analysis was performed using anti-Jra (A) or anti-Ebi (B).
(TIF)

Figure S5 **Genetic interaction between** ***ebi*** **and** ***thread*** **.** Scanning electron microscope analysis of *GMR-ebiΔC/+; +/+* (*ebiΔC; +/+*) or *GMR-ebiΔC/+; th^4^/+* (*ebiΔC; th^4^/+*).
(TIF)

Figure S6 **Light-dependent retinal degeneration in** ***GMR-ebiΔC*** **eyes.** *GMR-ebiΔC/+* (*ebiΔC*) ommatidia. (A) After 2 weeks of normal light conditions (12-h light/12-h dark), phenotype of the mutant retinae was mild. (B, C) Eyes of mutant flies cultured under constant illumination. The photoreceptor cells were retained after 1 week (B) but showed the severe degeneration phenotype after 2 weeks (C). (D) In contrast, the degeneration phenotype was suppressed by raising flies under constant darkness for 5 weeks after eclosion (compare with Figure 1C).
(TIF)

Methods S1 Oligonucleotides for RT-PCR and dsRNA.
(DOC)
