# Supplementary figures and images for: Ebi/AP-1 Suppresses Pro-Apoptotic Genes Expression and Permits Long-Term Survival of Drosophila Sensory Neurons
Source: PLoS One. 2012 May 30;7(5):e37028. doi: 10.1371/journal.pone.0037028 (PMC3364243; doi:10.1371/journal.pone.0037028)

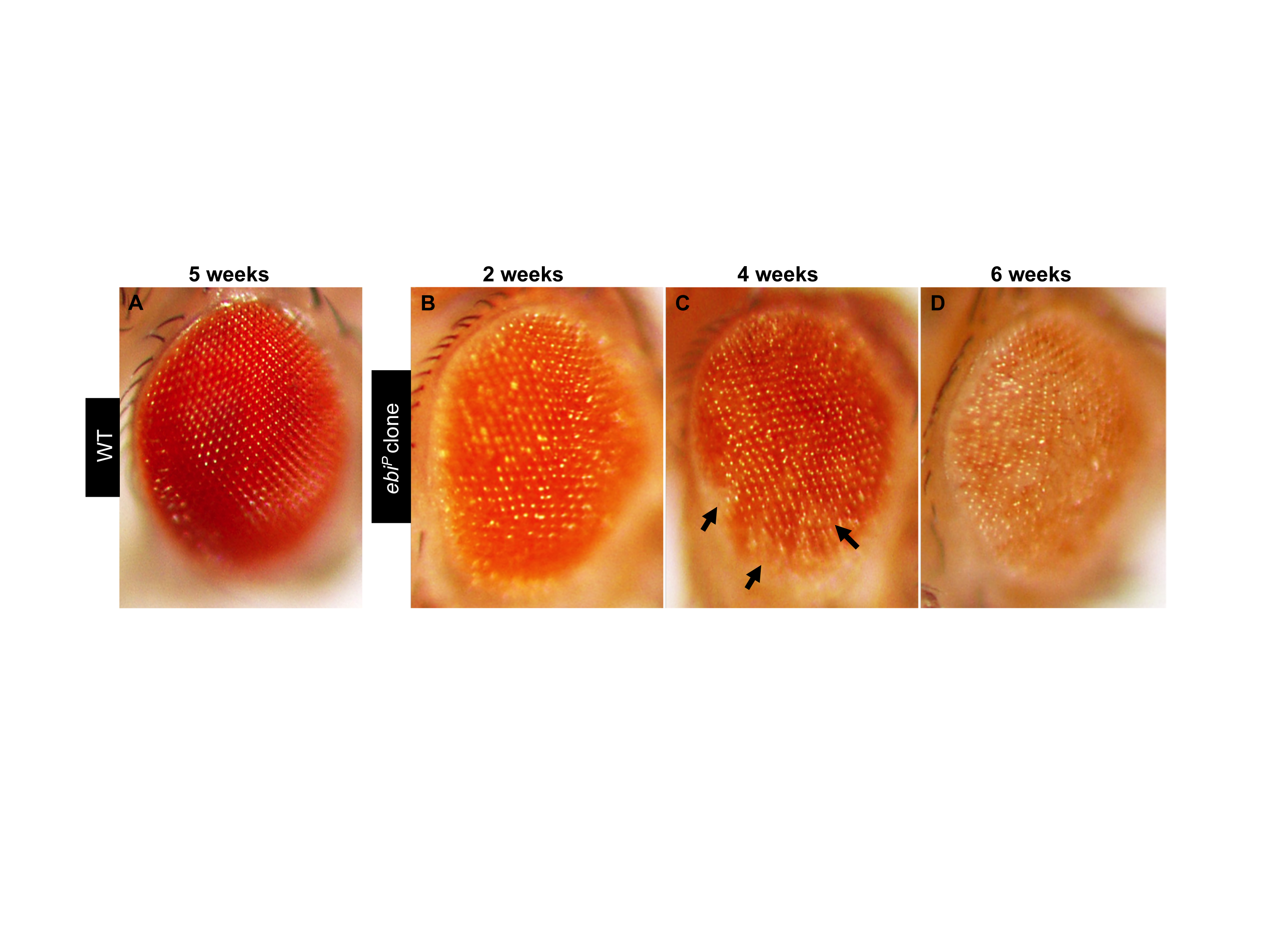

Supplement: Figure S1 — Age-dependent pigment loss in ebi mutant eyes. (A) Wild-type adult fly 5 weeks after eclosion. (B–D) ey-FLP; ebiP, FRT40A/CycEAR95, FRT40A (ebiP clone). After 2 weeks, eye pigment was retained (B). After 4 weeks, however, eye pigment was reduced (arrows; C). Severe pigment loss was observed after 6 weeks (D). (TIF) [file pone.0037028.s001.tif]

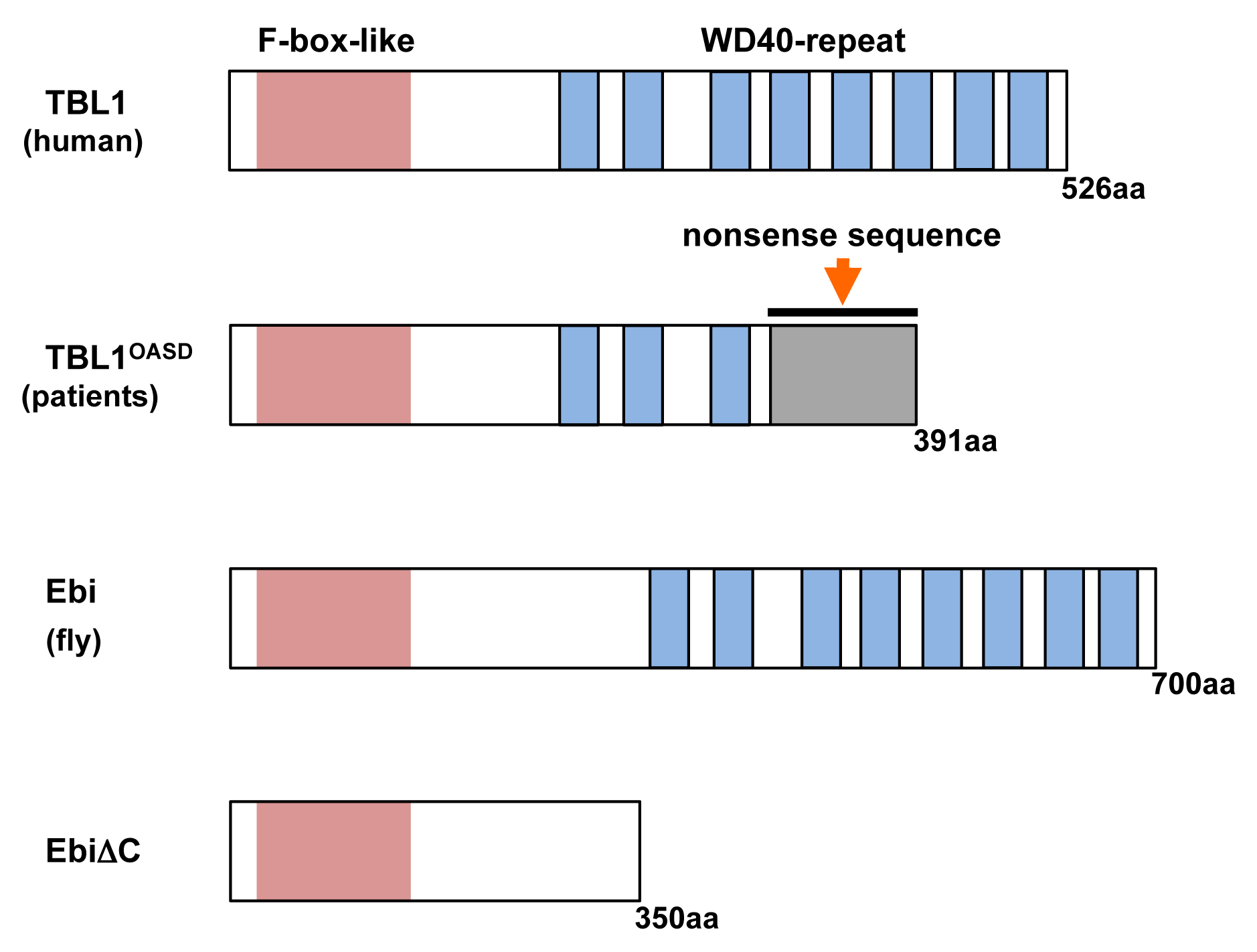

Supplement: Figure S2 — Structures of wild-type and mutant versions of TBL1 and Ebi. TBL1 from patients with OASD is truncated (TBL1OASD) because of a small deletion in the genomic DNA [17]. EbiΔC is a truncated form of Ebi that results in the deletion of the C-terminal WD40 repeats [13]. (TIF) [file pone.0037028.s002.tif]

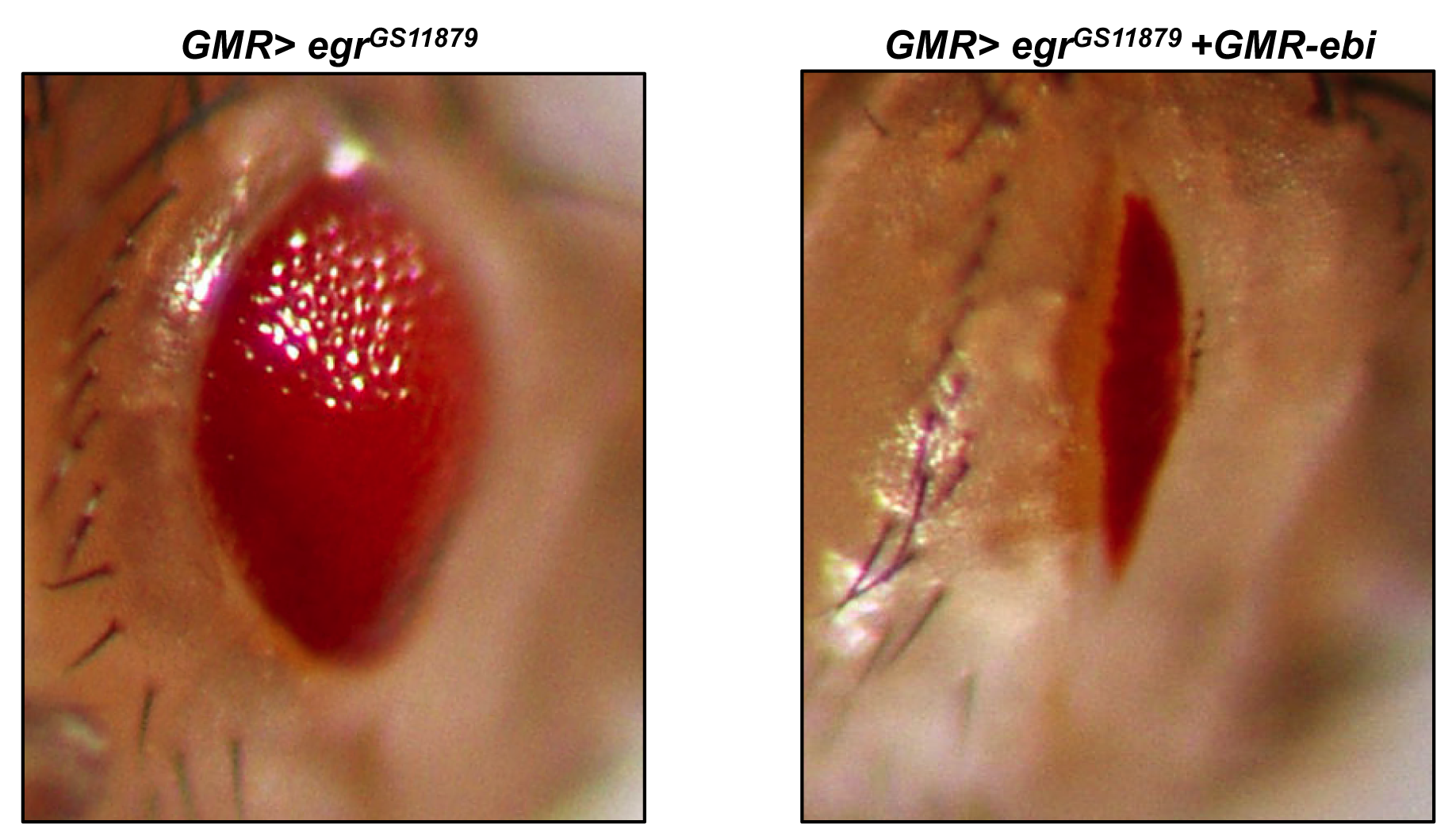

Supplement: Figure S3 — Overexpression of ebi enhanced egr induced small eye phenotype. GMR-Gal4/+; egrGS11687/+, TNF-α overexpression induced a small-eye phenotype. GMR-Gal4/+; egrGS11687/GMR-ebi, in which ebi was overexpressed under glass promoter, enhanced the eye phenotype. (TIF) [file pone.0037028.s003.tif]

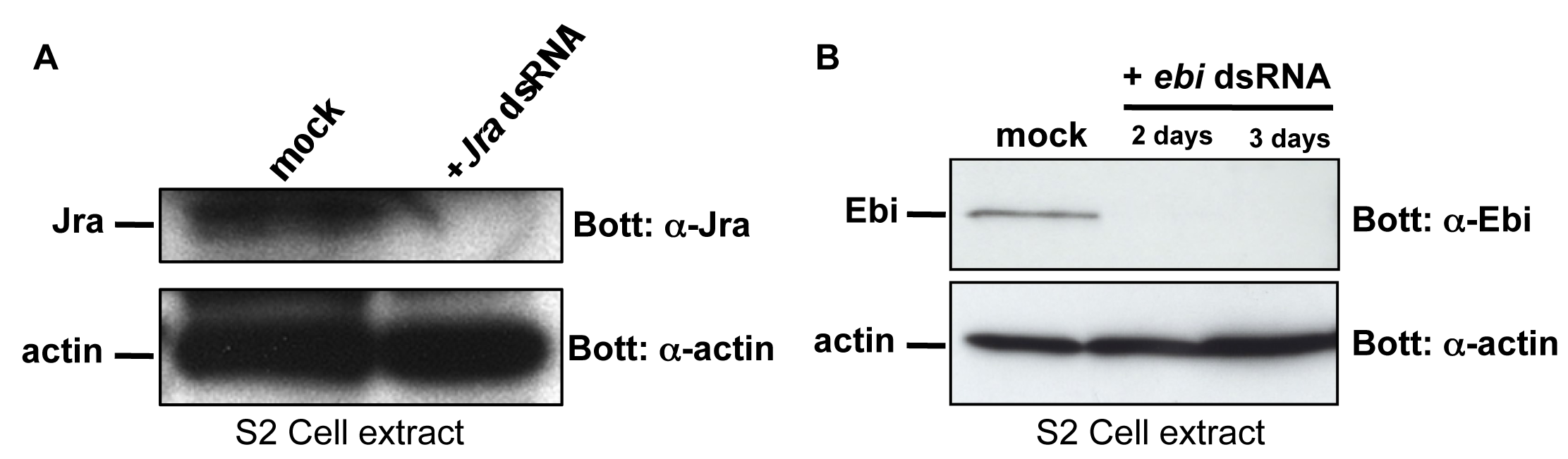

Supplement: Figure S4 — The efficiency of knock-down in Jra and ebi RNAi experiment. Western blot analysis was performed using anti-Jra (A) or anti-Ebi (B). (TIF) [file pone.0037028.s004.tif]

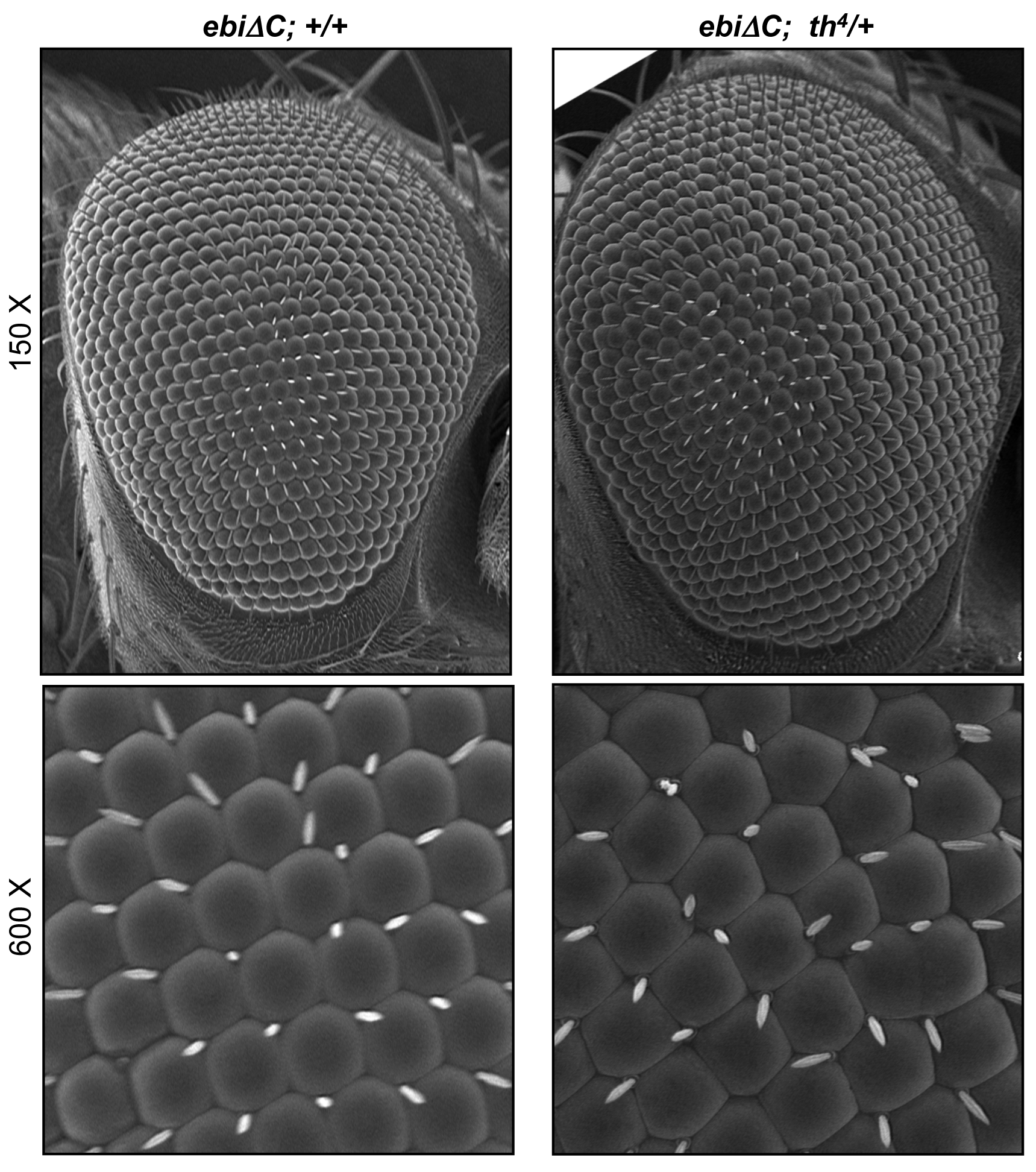

Supplement: Figure S5 — Genetic interaction between ebi and thread . Scanning electron microscope analysis of GMR-ebiΔC/+; +/+ (ebiΔC; +/+) or GMR-ebiΔC/+; th4/+ (ebiΔC; th4/+). (TIF) [file pone.0037028.s005.tif]

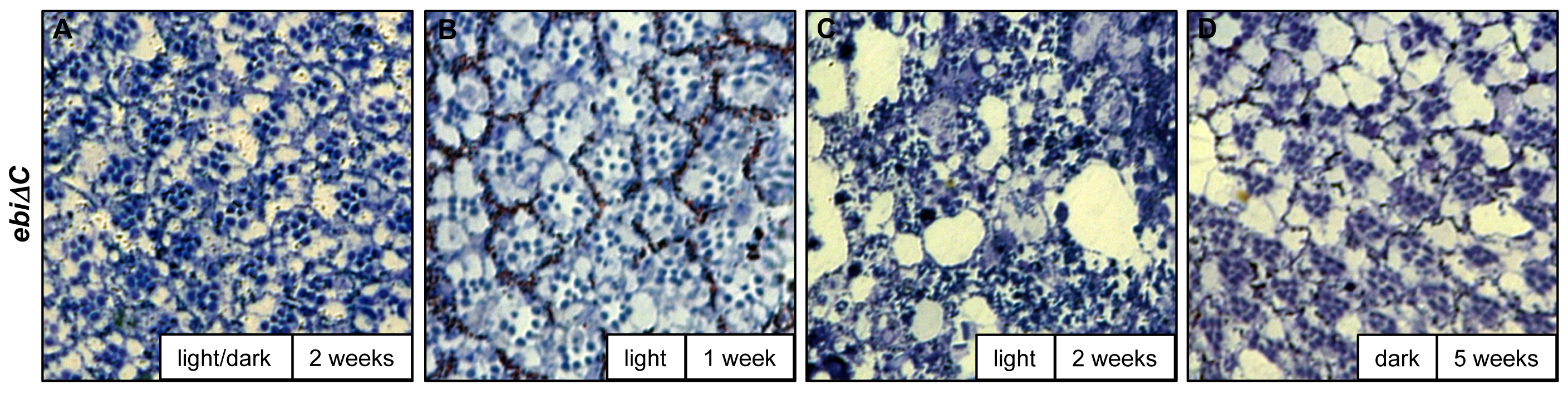

Supplement: Figure S6 — Light-dependent retinal degeneration in GMR-ebiΔC eyes. GMR-ebiΔC/+ (ebiΔC) ommatidia. (A) After 2 weeks of normal light conditions (12-h light/12-h dark), phenotype of the mutant retinae was mild. (B, C) Eyes of mutant flies cultured under constant illumination. The photoreceptor cells were retained after 1 week (B) but showed the severe degeneration phenotype after 2 weeks (C). (D) In contrast, the degeneration phenotype was suppressed by raising flies under constant darkness for 5 weeks after eclosion (compare with Figure 1C). (TIF) [file pone.0037028.s006.tif]
