# Supplementary material for: Ebi/AP-1 Suppresses Pro-Apoptotic Genes Expression and Permits Long-Term Survival of Drosophila Sensory Neurons
Source: PLoS One. 2012 May 30;7(5):e37028. doi: 10.1371/journal.pone.0037028 (PMC3364243; doi:10.1371/journal.pone.0037028)
Supplement: Methods S1 — Oligonucleotides for RT-PCR and dsRNA. (DOC) [file pone.0037028.s007.doc]

**Supplementary Information**

**Supplementary Materials**

***Oligonucleotides for RT-PCR and dsRNA***

Primers for the real-time PCR analysis and dsRNA were as follows:

For RT-PCR:

*hid*:

sense 5′-TGC GAA ATA CAC GGG TTC A-3′

antisense 5′-CCA ATA TCA CCC AGT CCC G-3′

*grim*:

sense 5′-TAG ATT CGC AGA GCG TAG CA-3′

antisense 5′-AAC AAT CGC AAC AAC AGC AG-3′

*reaper*:

sense 5′-ACG GGG AAA ACC AAT AGT CC-3′

antisense 5′-TGG CTC TGT GTC CTT GAC TG-3′

*sickle*:

sense 5′-CTT TGA AGG ACC TCC GTC TG-3′

antisense 5′-ATT GCT TCA GGA CCT TGC AC-3′

*puckered*:

sense 5′-CGA GGA TGG GTT TGA TTA CGA-3′

antisense 5′-TCA GTC CCT CGT CAA ATT GCT-3′

*rp49*:

sense 5′-GCT AAG CTG TCG CAC AAA TG-3′

antisense 5′-GTT CGA TCC GTA ACC GAT GT-3′

For RNAi

*Jra*:

sense 5′- CAT TTC CGT CCG CCA ATT CC-3′

antisense 5′-GTG CTC CAT CAC CTG CTG CTT C-3′

*ebi*:

sense 5′-ATG AGT TTT TCC AGC GAC GAG-3′

antisense 5′-CTC CAT TGT TTC CGG CGT ATG-3′
